# Early and long-term outcomes following redo mitral valve surgery in patients with prior minimally invasive mitral valve surgery

**DOI:** 10.1093/icvts/ivae042

**Published:** 2024-03-18

**Authors:** Katja Schumacher, Mateo Marin Cuartas, Manuela de la Cuesta, Thilo Noack, Philipp Kiefer, Sergey Leontyev, Michael A Borger, Marcel Vollroth, Martin Misfeld

**Affiliations:** University Department of Cardiac Surgery, Leipzig Heart Center, Leipzig, Germany; University Department of Cardiac Surgery, Leipzig Heart Center, Leipzig, Germany; University Department of Cardiac Surgery, Leipzig Heart Center, Leipzig, Germany; University Department of Cardiac Surgery, Leipzig Heart Center, Leipzig, Germany; University Department of Cardiac Surgery, Leipzig Heart Center, Leipzig, Germany; University Department of Cardiac Surgery, Leipzig Heart Center, Leipzig, Germany; University Department of Cardiac Surgery, Leipzig Heart Center, Leipzig, Germany; University Department of Cardiac Surgery, Leipzig Heart Center, Leipzig, Germany; University Department of Cardiac Surgery, Leipzig Heart Center, Leipzig, Germany; Department of Cardiothoracic Surgery, Royal Prince Alfred Hospital, Sydney, NSW, Australia; Sydney Medical School, University of Sydney, Sydney, NSW, Australia; Institute of Academic Surgery, Royal Prince Alfred Hospital, Sydney, Australia; The Baird Institute of Applied Heart and Lung Surgical Research, Sydney, Australia

**Keywords:** Mitral valve surgery, Redo surgery, Minimally invasive surgery

## Abstract

**OBJECTIVES:**

The frequency of minimally invasive mitral valve surgery (MVS) has steadily increased over the last decades and therefore surgeons are now encountering an increasing number of patients requiring mitral valve (MV) reoperations post-minimally invasive MVS. The aim of this study was to analyse the early postoperative outcomes and the long-term survival in patients who undergo reoperative MVS following previous minimally invasive surgery.

**METHODS:**

Patients who underwent redo MVS following prior minimally invasive MVS between January 2002 and December 2021 were included in our analysis. Study data were prospectively collected and retrospectively analysed. The primary outcomes were 30-day mortality and long-term survival.

**RESULTS:**

Among the 187 included patients, 34 (18.2%) underwent repeat MV repair and 153 (81.8%) MV replacement. The median age was 66 years (interquartile range 56–74) and 80 (42.8%) patients were female. Redo MVS was performed through median sternotomy in 169 patients (90.4%). A total of 77 (41.2%) patients had additional concomitant procedures. The median intensive care unit stay was 1 day (1–5). The 30-day mortality was 6.4% (12/187). Estimated survival at 5 and 12 years was 61.8% and 38.3%, respectively. Preoperative stroke (hazard ratio 3.28, 95% confidence interval 1.37–7.85, *P* = 0.007) as well as infective endocarditis (hazard ratio 1.85; 95% confidence interval 1.09-3.11, *P* = 0.021) were independent predictors of long-term mortality.

**CONCLUSIONS:**

Redo MVS following prior minimally invasive MVS can be performed safely with low early perioperative mortality and acceptable long-term survival. Preoperative stroke, infective endocarditis and concomitant tricuspid valve surgery are independent predictors of long-term mortality.

**Figure:**
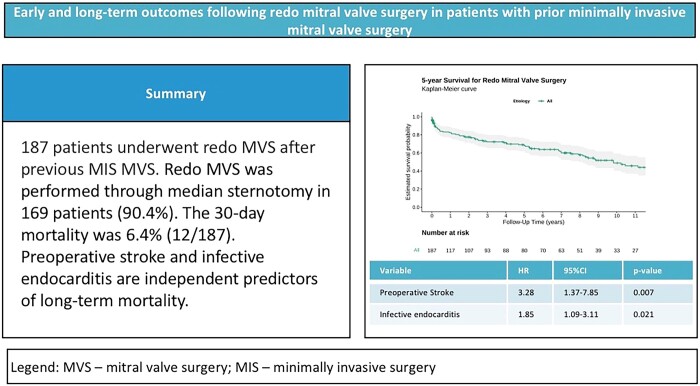

## INTRODUCTION

The number of patients requiring reoperative mitral valve surgery (MVS) has increased over the past decades due to more frequent implantation of bioprosthetic valves, the steady increase in prosthetic valve endocarditis, as well as the ageing population [1]. Reoperation rates up to 35% are reported in current series of patients undergoing MVS [2]. Reoperations are technically more demanding due to mediastinal and pericardial adhesions increasing the risk of injuring cardiac and mediastinal structures. Furthermore, patients requiring redo MVS often present with more comorbidities [3]. These factors explain why redo MVS is associated with higher mortality and morbidity than primary procedures [4, 5].

In Germany, isolated MVS is performed more often using a minimally invasive surgery (MIS) approach (53.5%) than through median sternotomy [6]. Primary MIS MVS could lower the risks associated with subsequent redo MVS, since resternotomy is avoided and mediastinal adhesions may be reduced [3, 7]. However, there is a lack of data reporting on the outcomes of patients who underwent redo MVS after a previous MIS approach. Therefore, the aim of this study was to analyse the early and long-term outcomes following redo MVS in patients who previously underwent MIS MVS. We hypothesize that redo MVS in this specific subset of patients can be performed safely and with good early and long-term outcomes.

## PATIENTS AND METHODS

### Ethical statement

This research project was approved by the ethics committee from the University of Leipzig (Protocol number 476/19-ek, date of approval 18 December 2019). Individual patient informed consent was waived due to the anonymous data management and the retrospective nature of this study.

### Study cohort

A total of 187 consecutive patients who underwent redo MVS following previous MIS MVS between January 2002 and December 2021 were included in our analysis. We included all patients undergoing redo MVS after previous MIS MVS regardless of concomitant procedures or urgency of the surgery. Preoperative patient characteristics, intraoperative data and postoperative outcomes were prospectively collected and entered into a computerized database and were retrospectively analysed. Primary study outcomes were 30-day mortality and long-term survival. Secondary end points were early postoperative complications.

### Surgical technique

Intraoperative access was achieved in 90% of all cases through a median sternotomy using aorto-bicaval cannulation. In cases of minimally invasive thoracotomy through the 4th intercostal space, cannulation of the femoral vessels was performed. Crystalloid cardioplegia was administered and repeated after 90–120 min, if necessary. Surgery was performed at a moderate hypothermia of 34°C. Mitral valve (MV) access was obtained through left atrium incision. After inspection of the MV anatomy, either repair or replacement was performed.

According to the current guidelines, patients with MV repair as well as biological prostheses received oral anticoagulation with vitamin K antagonists for 3 months and patients with mechanical prostheses received life-long anticoagulation.

### Follow-up

Patient demographics and surgical data were obtained from the medical records of the Leipzig Heart Center. Follow-up data were collected during subsequent visits in our outpatient clinic and by mailed questionnaires or phone contact. Follow-up was closed on 2 July 2023.

### Statistical methods

Categorical variables are expressed as frequencies and percentages throughout the manuscript. Continuous variables are expressed as mean standard deviation for normally distributed variables and median and interquartile range (IQR) for non-normally distributed variables. Normalcy of distribution of all variables was tested with the Shapiro–Wilk test. Long-term survival was estimated using the Kaplan–Meier method. To determine clinical predictors of long-term mortality, multivariate Cox proportional hazards regression models were performed. Perioperative variables that had a univariable value of *P *<* *0.2 or those judged to be clinically important were submitted to a multivariable Cox proportional hazard model by backward stepwise selection. The results of the Cox regression model are reported as hazard ratios with 95% confidence interval. The method used to compare mortality between 2 groups was Fisher’s exact test. Statistical analyses were performed using the SPSS software package, version 25.0 (IBM Corp, Armonk, NY, USA) and Microsoft Excel 2019 for Mac.

## RESULTS

### Patient baseline characteristics

Among the 187 enrolled patients, 131 (70.1%) underwent MIS MV repair during the index procedure and 56 (29.9%) MIS MV replacement. The median elapsed time between first MVS and redo MVS was 18.4 (IQR 5—62.3) months. Recurrent mitral regurgitation was the most common indication for reoperation (*n* = 127, 67.9%), while infective endocarditis was the second most common surgical indication (*n* = 49; 26.2%). MV stenosis and thrombosis were both rare with 5 cases each (2.7%). Median age at reoperation was 66 years (IQR 56–74 years) and 80 patients (42.8%) were female. Further baseline characteristics are shown in Table 1.

**Table 1:** Baseline characteristics prior to redo surgery

	*N* = 187
Age at first surgery (years)	60.5 (53–69)
Age at second surgery (years)	66 (56–74)
Weight (kg)	76.2 (67–88)
Height (cm)	172 (164–180)
BMI (kg/cm^2^)	25.2 (23–28.9)
Female	80 (42.8)
Coronary artery disease	30 (16.0)
Previous myocardial infarction	10 (5.3)
Previous PCI	16 (8.6)
COPD	24 (12.8)
Pulmonary hypertension	70 (37.4)
Atrial fibrillation	111 (59.4)
Previous pacemaker implantation	29 (10.7)
Arterial hypertension	134 (71.7)
Diabetes mellitus	43 (23.0)
Chronic kidney disease	56 (30.0)
Previous dialysis	5 (2.7)
Previous stroke	13 (7.0)
Peripheral arterial disease	7 (3.7)
Preoperative EF (%)	55 (45–64)
EuroSCORE II	5.62 (3.02–13.98)
Preoperative mitral regurgitation severity	
None	36 (19.3)
Mild	26 (13.9)
Moderate	46 (24.5)
Moderate to severe	77 (41.2)
Severe	2 (1.1)
Preoperative mitral stenosis severity	
None	151 (80.7)
Mild	14 (7.5)
Moderate	11 (5.9)
Moderate to severe	11 (5.9)
Previous surgery	
MV repair	131 (70.1)
MV replacement	56 (29.9)
Indication for first MVS	
Anterior leaflet prolaps	39
Posterior leaflet prolaps	50
Bileaflet prolapse	21
Endocarditis	13
LA dilatation due to atrial fibrillation	19
Dilatative cardiomyopathy	8
Ischaemic cardiomyopathy	7
Mitral valve stenosis	5
Combined stenosis/regurgitation	18
Other	7

Continuous variables are expressed as median and interquartile range in parentheses. Categorical variables are expressed as numbers (*n*) and percentages in parentheses.

BMI: body mass index; COPD: chronic obstructive pulmonary disease; EF: ejection fraction; MV: mitral valve; MVS: mitral valve surgery; PCI: percutaneous coronary intervention.

### Intraoperative variables

Intraoperative details are presented in Table 2. A total of 103 patients (55.1%) underwent isolated redo MVS, and 84 (44.1%) underwent redo MVS plus concomitant cardiac surgical procedures. Redo surgery was performed through a median sternotomy in 169 patients (90.4%). Intraoperative extracorporeal life support implantation was required in 2 patients (1.1%) due to failed weaning from cardiopulmonary bypass, while implantation of an intra-aortic balloon pump was required in 3 patients (1.6%). Intraoperative mortality was 0.5% (1/187 patients).

**Table 2:** Intraoperative details

Indication for redo surgery	
MV regurgitation	127 (67.9)
Endocarditis	49 (26.2)
MV stenosis	5 (2.7)
MV thrombosis	5 (2.7)
Surgical approach	
Sternotomy	169 (90.4)
Right mini-thoracotomy	18 (9.6)
CPB time (min)	105 (83–133)
Aortic cross-clamp time (min)	68 (53–84)
Surgical procedure	
MV repair	34 (18.2)
MV replacement	153 (81.8)
Valve type	
SJM Epic	91 (59.4)
Medtronic ATS	21 (13.7)
Carpentier Edwards Perimount Mitral	10 (6.5)
Edwards SAPIEN III	1 ((0.7)
Medtronic Mosaic	27 (17.6)
Medtronic Hancock II	2 (1.4)
BioIntegral Stentless prosthesis	1 (0.7)
Additional procedures	
Tricuspid valve repair	44 (23.5)
Tricuspid valve replacement	9 (4.8)
ASD closure	4 (2.1)
CABG	6 (3.2)
Aortic valve surgery	17 (9.1)
LAA closure	7 (3.7)
Cryoablation	18 (9.6)
Supracommisural ascending aorta replacement	1 (0.5)
Intraoperative ECLS	2 (1.1)
Intraoperative IABP	3 (1.6)
Timing of surgery	
Elective	95 (50.8)
Urgent	68 (36.4)
Emergency	24 (12.8)

Continuous variables are expressed as median and interquartile range in parentheses. Categorical variables are expressed as numbers (*n*) and percentages in parentheses.

ASD: atrial septal defect; CABG: coronary artery bypass grafting; CPB: cardiopulmonary bypass time; ECLS: extracorporal life support; IABP: intra-aortic balloon pump; LAA: left atrial appendage; MV: mitral valve.

### Early postoperative outcomes

Thirty-day mortality was 6.4% (12/187 patients). Patients with endocarditis represented 83.3% of the patients who died within the first 30 days (10/12 patients), and in-hospital mortality was significantly lower in non-endocarditis patients [22.4% (11/49) vs 2.9% (4/138) *P* < 0.01]. The median intensive care unit stay was 1 day (IQR 1–5 days). Re-exploration for bleeding was required in 25 patients (13.4%). A total of 15 patients (8.0%) developed postoperative low cardiac output syndrome. Among them, 6 patients (3.2%) with severe postcardiotomy cardiogenic shock required mechanical circulatory support [3 patients (1.6%) extracorporeal life support and 3 patients (1.6%) intra-aortic balloon pump]. Further postoperative outcomes are summarized in Table 3.

**Table 3:** Postoperative outcomes

Postoperative complications	
Stroke	7 (3.7)
Myocardial infarction	3 (1.6)
ECMO	3 (1.6)
IABP	3 (1.6)
Low cardiac output syndrome	15 (8.0)
Pacemaker	22 (11.8)
Bleeding requiring re-exploration	25 (13.4)
Dialysis	33 (17.6)
Superficial wound healing impairment	6 (3.2)
Postoperative outcomes	
30-Day mortality (%)	6.4
ICU stay (days)	1 (1–5)
Hospital stay (days)	14 (9–24)
Residual MR	27 (14.4)
I	24 (12.8)
II	3 (1.6)
Paravalvular leak	6 (3.2)

Continuous variables are expressed as median and interquartile range in parentheses. Categorical variables are expressed as numbers (n) and percentages in parentheses.

ECMO: extracorporeal membrane oxygenation; IABP: intra-aortic balloon pump; ICU: intensive care unit; MR: mitral regurgitation.

### Follow-up

**Figure 1:**
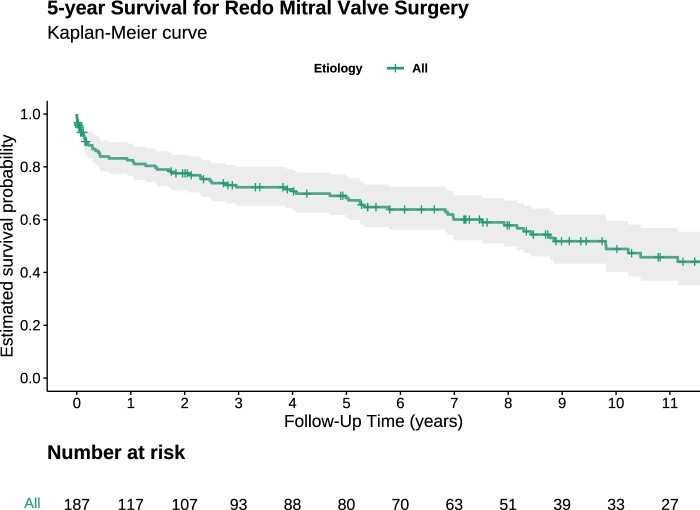
Kaplan–Meier estimate of long-term survival following redo mitral valve surgery after minimally invasive mitral valve surgery.

**Figure 2:**
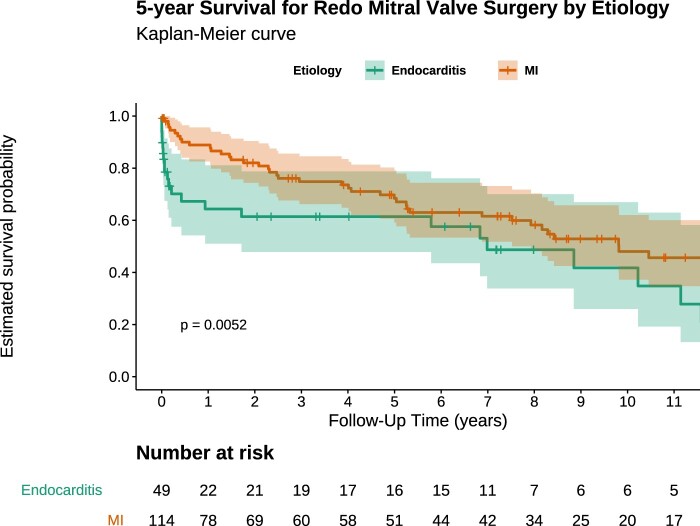
Kaplan–Meier estimate of long-term survival following redo mitral valve surgery after minimally invasive mitral valve surgery stratified by aetiology.

Estimated survival at 5 and 12 years was 61.8% and 38.3%, respectively (Fig. 1). Patients presenting with infective endocarditis had worse survival than patients presenting with MV regurgitation (Fig. 2). Preoperative stroke and infective endocarditis were identified as independent predictors of long-term mortality (Table 4). The type of previous MV operation (i.e. MV repair or replacement) was not identified as an independent predictor of long-term mortality following redo MVS (hazard ratio 0.56; 95% confidence interval 0.2–1.4; *P* = 0.23).

**Table 4:** Predictors of long-term mortality

Variable	Univariate			Multivariate		
	HR	CI	*P*-value	HR	CI	*P*-value
Gender	0.99	0.62–1.59	0.993			
Coronary artery disease	1.47	0.82–2.63	0.197	1.33	0.73–2.41	0.354
COPD	1.52	0.82–2.83	0.185	1.48	0.77–2.85	0.243
Myocardial infarction	0.94	0.38–2.33	0.893			
Pulmonary hypertension	0.94	0.58–1.52	0.787			
Arterial hypertension	0.99	0.60–1.59	0.929			
Atrial fibrillation	0.976	0.61–1.55	0.919			
Diabetes	1.73	1.05–2.85	0.030	1.49	0.87–2.55	0.150
Chronic kidney disease	1.78	1.09–2.90	0.022	1.05	0.59–1.86	0.855
Endocarditis	2.23	1.37–3.63	0.001	1.85	1.09–3.11	0.021
Mitral valve insufficiency	0.79	0.49–1.25	0.308			
Previous stroke	3.46	1.63–7.37	0.001	3.28	1.37–7.85	0.007

CI: confidence interval; COPD: chronic obstructive pulmonary disease; HR: hazard ratio.

## DISCUSSION

The current study represents a single-centre experience over a period of 19 years in patients undergoing redo MVS following prior MIS MVS. The main findings of our study are:

1. Recurrent MV regurgitation (67.9%) and infective endocarditis (26.2%) were the most common indications for reoperation.
2. Thirty-day mortality was 6.4% (12/187 patients). The majority of patients that died within the first 30 postoperative days presented with endocarditis (83.3% 10/12 patients).
3. Early severe postoperative complication rates were low, considering the redo setting and the high-risk profile of these patients. In addition, the median intensive care unit stay was short at only 1 day.
4. Long-term survival was acceptable, considering the higher operative risk associated with reoperations and the high incidence of endocarditis in our patient population.
5. Preoperative stroke, infective endocarditis as well as concomitant tricuspid valve surgery were significant predictors for long-term mortality after redo MVS.

Although the role of transcatheter valve implantation has gained importance over the past decades, conventional surgery remains the gold standard in patients requiring MV redo procedures and the only option in patients presenting with endocarditis [8]. There are several single and multicentre studies describing the outcomes after redo MVS in different patient cohorts [4, 5, 9–11]. However, due to selection bias in many of these cohorts (e.g. exclusion of patients with endocarditis), the results are inconsistent and therefore difficult to compare between series. Furthermore, studies presenting the outcomes of redo surgery after prior MIS MVS are lacking. In the present study, we aimed to present a real-life cohort of all patients undergoing redo MVS following prior MIS MVS at our institution, regardless of the surgical indication or concomitant procedures.

Previous studies have demonstrated a wide range of 30-day mortality rates for redo MVS, ranging from 1.3% to 22.4% [2, 10]. Although most of these cohorts include patients with both MIS approach and sternotomy during the first MVS, sternotomy predominates, representing over 80% of patients [5, 9, 10]. Mehaffey *et al.* [4] reported a 30-day mortality of 11.1% analysing a large cohort (*n* = 1096) of patients with non-infective as well as infective MV disease undergoing redo MVS with concomitant procedures. In contrast, Speiser *et al.* [11] demonstrated a 30-day mortality of 6.9% in patients undergoing redo isolated MVS following prior MVS via sternotomy. The lower early mortality of this cohort is at least partially explained by the exclusion of all patients with infective endocarditis and patients who underwent concomitant procedures [11]. These studies emphasize the effect of patient selection on reported outcomes, although both studies report on the same surgical approaches.

Due to the increasing number of MIS MVS being performed over time [6], it is important to evaluate the risks of redo MVS after a previous MIS approach. The observed 30-day mortality (6.4%) in our cohort suggests that that redo MVS following MIS MVS can be performed safely, even in higher-risk patients. After excluding patients operated for infective endocarditis, the 30-day mortality rate was even more notable (2.9%). One patient who died during surgery presented with infective endocarditis of the mitral as well as aortic valve and underwent surgery in an already critical preoperative state.

There are several risk factors described for increased mortality and major adverse events after redo MVS. In our patient population, preoperative stroke, infective endocarditis and concomitant tricuspid valve surgery were significant independent predictors of increased long-term mortality following redo MVS. Other studies have also shown that prior myocardial infarction, pulmonary hypertension and cardiogenic shock have also been reported as independent predictors [4, 11]. Certain risk factors such as preoperative comorbidities or required concomitant procedures are not modifiable. However, the occurrence of postoperative end-organ complications caused by potentially preventable events has been demonstrated to be independent predictor of postoperative mortality [12]. Iatrogenic lesions during mediastinal re-entry or injury of a previous patent left internal mammary graft leading to excessive blood loss is associated with a 4-fold increase in postoperative mortality [12]. This correlation emphasizes the importance of adequate surgical planning prior to redo MVS. Primary MIS MVS might decrease the risk of postoperative bleeding and catastrophic injury of vital structures, since resternotomy is avoided and mediastinal adhesions are generally less [3, 7]. Furthermore, prolonged operating and cardiopulmonary bypass times as well as suboptimal myocardial protection represent potential preventable risk factors for increased perioperative mortality [10, 12]. In addition to reducing mortality by addressing preoperative predictors, future repeat redo procedures should be avoided as well. In this regard, proper surgical technique and surgical decision-making should be encouraged, especially in patients undergoing redo MV repair [13–15].

The number of MV-in-valve and valve-in-ring transcatheter procedures for recurrent MV disease are increasing and may be an attractive option to redo MVS. However, certain indications such as endocarditis remain treatable only with conventional surgery. Moreover, redo MVS has shown to be superior to transcatheter approaches after failed surgical MV repair [16]. Additionally, in contrast to transcatheter approaches, redo surgery enables treatment of concomitant conditions, such as atrial fibrillation or tricuspid valve regurgitation. Furthermore, there is no significant difference in mortality between redo MVS and valve-in-valve procedures [16, 17]. Finally, transcatheter MV-in-ring procedures have been associated with disappointing results [18]. When evaluating a patient requiring a redo mitral intervention after prior MVS, it is important to note that the risk of redo surgery has dramatically decreased over time and it is currently significantly better than predicted by risk scores [4]. For all of these reasons, it is reasonable to assume that redo MVS will remain an important surgical option for appropriate patients for many years to come. The performance of previous MVS through a MIS approach rather than through a sternotomy may be another method of improving the results for redo MVS surgery.

### Limitations

There are several limitations of this study that should be noted. First, this is a single-centre retrospective study without a control group with the corresponding limitations associated with its nature. Moreover, the study does not compare different surgical and interventional strategies. Furthermore, given the long-term retrospective nature of the study and, therefore, markedly differing follow-up periods among patients and a limited cohort size, more complex statistical analyses were not feasible. Our methods to follow-up on survival was limited to visits at our outpatient clinic as well as phone calls. Since the majority of patients were monitored by referring cardiologists, we cannot provide any data presenting long-term echocardiographic parameters. Finally, we tried to present a real-life cohort undergoing MVS after previous MIS MVS so the included patients differ in many aspects such as comorbidities, concomitant procedures as well as used prostheses.

## CONCLUSION

Although being a technically challenging procedure, redo MVS following prior minimally invasive MVS can be performed safely with low early postoperative mortality and acceptable long-term survival. Preoperative stroke, infective endocarditis as well as concomitant tricuspid valve surgery were significant predictors of increased long-term mortality in such patients.

**Conflict of interest:** Michael A. Borger discloses that his hospital receives speakers’ honoraria and/or consulting fees on his behalf from Edwards Lifesciences, Medtronic, Abbott and CryoLife. The remaining authors have no conflicts of interest or financial relationships with the industry to disclose.

## Data Availability

The data underlying this article will be shared upon request to the corresponding author.

## ABBREVIATIONS

IQR: Interquartile range
MIS: Minimally invasive surgery
MV: Mitral valve
MVS: Mitral valve surgery
